# High blood pressure and associated factors among HIV-infected young persons aged 13 to 25 years at selected health facilities in Rwenzori region, western Uganda, September–October 2021

**DOI:** 10.1186/s40885-022-00230-5

**Published:** 2023-04-15

**Authors:** Richard Migisha, Alex Riolexus Ario, Daniel Kadobera, Lilian Bulage, Elizabeth Katana, Alex Ndyabakira, Peter Elyanu, Julius N. Kalamya, Julie R. Harris

**Affiliations:** 1 Public Health Fellowship Program, Kampala, Uganda; 2 Ministry of Health, Kampala, Uganda; 3 Baylor College of Medicine Children’s Foundation-Uganda, Kampala, Uganda; 4 Division of Global HIV and TB, U.S. Centers for Disease Control and Prevention (CDC), Kampala, Uganda; 5 Division of Global Health Protection, Centers for Disease Control and Prevention, Kampala, Uganda

**Keywords:** Young adult, Adolescent, HIV, Hypertension, Prehypertension, Uganda

## Abstract

**Background:**

High blood pressure (HBP), including hypertension (HTN), is a predictor of cardiovascular events, and is an emerging challenge in young persons. The risk of cardiovascular events may be further amplified among people living with HIV (PLHIV). We determined the prevalence of HBP and associated factors among PLHIV aged 13 to 25 years in Rwenzori region, western Uganda.

**Methods:**

We conducted a cross-sectional study among PLHIV aged 13 to 25 years at nine health facilities in Kabarole and Kasese districts during September 16 to October 15, 2021. We reviewed medical records to obtain clinical and demographic data. At a single clinic visit, we measured and classified BP as normal (< 120/ < 80 mmHg), elevated (120/ < 80 to 129/ < 80), stage 1 HTN (130/80 to 139/89), and stage 2 HTN (≥ 140/90). We categorized participants as having HBP if they had elevated BP or HTN. We performed multivariable analysis using modified Poisson regression to identify factors associated with HBP.

**Results:**

Of the 1,045 PLHIV, most (68%) were female and the mean age was 20 (3.8) years. The prevalence of HBP was 49% (*n* = 515; 95% confidence interval [CI], 46%–52%), the prevalence of elevated BP was 22% (*n* = 229; 95% CI, 26%–31%), and the prevalence of HTN was 27% (*n* = 286; 95% CI, 25%–30%), including 220 (21%) with stage 1 HTN and 66 (6%) with stage 2 HTN. Older age (adjusted prevalence ratio [aPR], 1.21; 95% CI, 1.01–1.44 for age group of 18–25 years vs. 13–17 years), history of tobacco smoking (aPR, 1.41; 95% CI, 1.08–1.83), and higher resting heart rate (aPR, 1.15; 95% CI, 1.01–1.32 for > 76 beats/min vs. ≤ 76 beats/min) were associated with HBP.

**Conclusions:**

Nearly half of the PLHIV evaluated had HBP, and one-quarter had HTN. These findings highlight a previously unknown high burden of HBP in this setting’s young populations. HBP was associated with older age, elevated resting heart rate, and ever smoking; all of which are known traditional risk factors for HBP in HIV-negative persons. To prevent future cardiovascular disease epidemics among PLHIV, there is a need to integrate HBP/HIV management.

## Introduction

High blood pressure (HBP), including hypertension (HTN), is recognized as a leading predisposing factor for cardiovascular disease (CVD) [1]. While this has historically been recognized in high-resource settings, it is increasingly being recognized as a public health issue in low-resource settings [2]. While most cases of HTN are recognized among adults, it has its origins in the early stages of life, from late childhood or teenage years to later life [3]. In young persons, HTN is a growing problem globally, largely due to changes in lifestyle behaviors and the lowering of the diagnostic thresholds for HTN [4]. A recent study among young Ugandan adults (aged 18–40 years) reported a prevalence of HTN of 15%, with 40% of individuals being prehypertensive [5].

People living with HIV (PLHIV) with HBP have a significantly higher risk of cardiovascular events and mortality than HIV-uninfected persons with HBP, which is thought to be due to a complex interplay of various factors including immune dysfunction, endothelial dysfunction, and inflammatory changes [6–8]. In addition, some data suggest that individuals may develop CVD earlier in life than uninfected individuals [9–11]. However, the lack of robust data on the burden of noncommunicable diseases (NCDs) among HIV-infected persons in resource-limited settings presents a barrier to the planning for or implementation of large-scale NCD/HIV integrated care, including HTN prevention and management among PLHIV [12].

In Uganda, national guidelines for integration of screening and management of NCDs among PLHIV (regardless of age) exist [13]; however, just as in other sub-Saharan African settings, screening for HBP in young persons is often overlooked in routine clinical practice [14]. This may result in low rates of HBP diagnosis and delayed management, to improve cardiovascular outcomes in these young persons. In addition, although HBP has been widely studied in adults with HIV infection, few studies have been done to evaluate the prevalence of HBP and associated factors in young persons with HIV infection. Those that have been done have implicated HIV as a potential risk factor for early onset of HBP among adolescents and young adults [15, 16]. Assessing the prevalence and risk factors for HBP in young HIV-infected persons may inform risk mitigation measures. We determined the prevalence of and factors associated with HBP among adolescents and young persons with HIV infection aged 13 to 25 years in the Rwenzori region, western Uganda.

## Methods

### Ethics statement

Our study utilized secondary data routinely collected in antiretroviral therapy (ART) clinics. The BP measurements were part of routine good clinical practice at the ART clinics. We obtained written informed consent for persons aged 18 to 25 years and written informed assent for those aged 12 to 17 years before taking their BP measurements. In addition, the parents/guardians of the minors (< 18 years) provided written informed consent before participation. We did not capture any personal identifiers. We obtained permission to use the secondary data from the district health officers of Kasese and Kabarole districts and the directors of the respective health facilities. In addition, the US Centers for Disease Control and Prevention’s Center for Global Health determined our study was nonresearch whose primary intention was to address public health problems. The data were stored in password-protected computers and data was not shared with anyone outside the investigation team.

### Study design setting and population

We conducted a cross-sectional study among PLHIV aged 13 to 25 years at selected clinics supported by Baylor College of Medicine Children’s Foundation-Uganda (Baylor Uganda) in the Rwenzori region (Kasese and Kabarole districts). Baylor Uganda was the implementing partner supporting HIV and AIDS prevention, care, and treatment services in the region, funded by President’s Emergency Plan for AIDS Relief (PEPFAR), and also strengthened routing BP measurements in clinics providing ART in the region. The study utilized both primary and secondary data. We obtained laboratory, demographic, and clinical data through retrospective review of medical records and took BP measurements (see procedure below) for the participants at a single clinic visit at each of the nine clinics during the study period (September 16–October 15, 2021).

### Sample size and sampling considerations

**Fig. 1:**
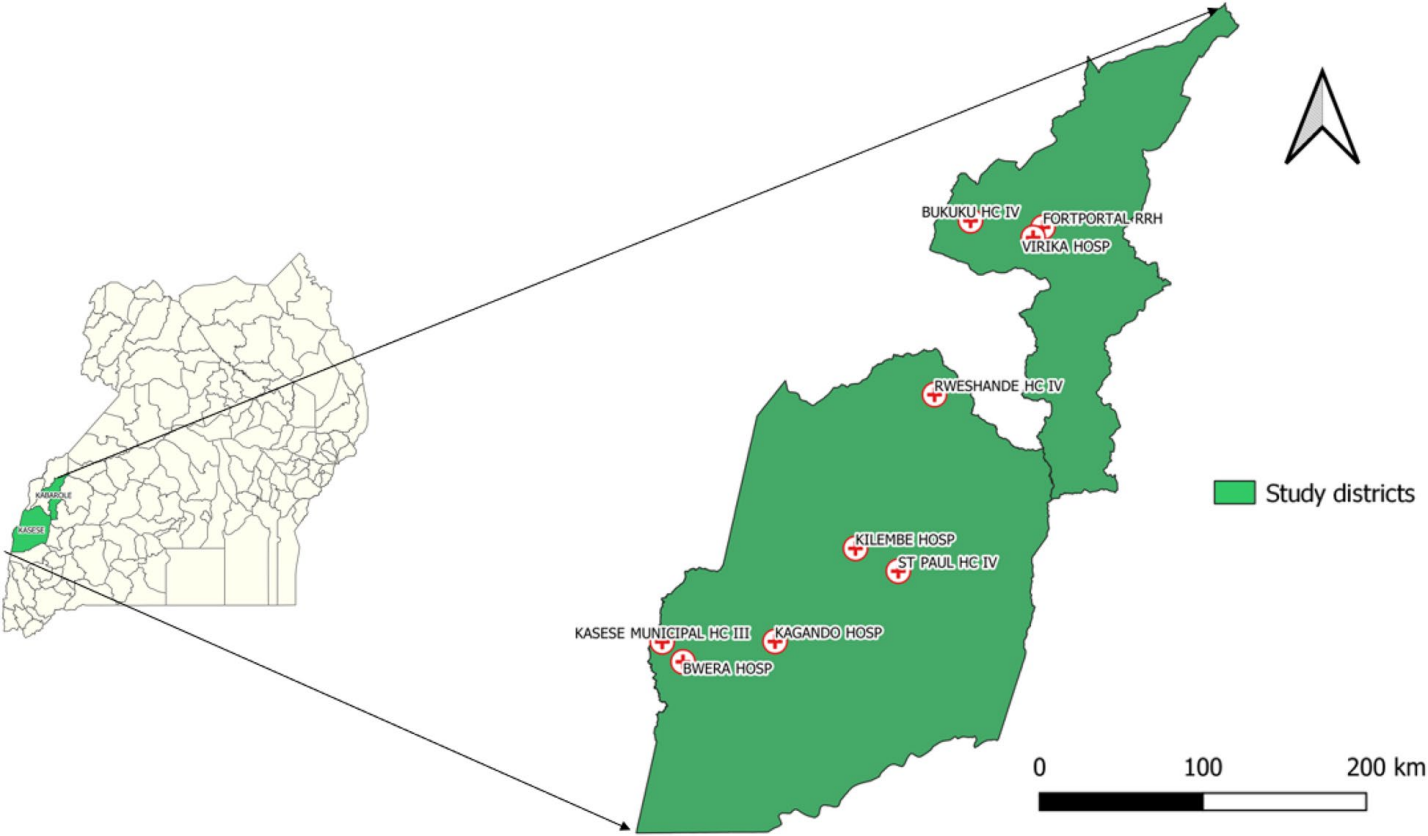
Selected health facilities for the study in Kabarole and Kasese districts, Rwenzori region, western Uganda, from September to October 2021. HC, Health Center; RRH, Regional Referral Hospital; HOSP, Hospital

We purposively selected nine health facilities in Kasese and Kabarole districts that had scheduled their adolescent clinic days within our study period (Fig. 1). Facilities selected in Kabarole District were Bukuuku Health Center IV, Virika Hospital, and Fort Portal Regional Referral Hospital. Facilities selected in Kasese District were Kagando Hospital, Kilembe Mines Hospital, Bwera Hospital, Rwesande HCIV, Kasese Municipal Health Center III, and St. Paul Medical Centre. We enrolled all 1,045 treatment-experienced young persons aged 13 to 25 years who came to refill antiretroviral drugs (ARV) within the study period.

### Study variables and data collection

Clinic nurses trained in the use of a standardized data collection tool abstracted data from study participants’ medical records on demographics (age and sex) and clinical data (weight, height, BP, mode of transmission and duration of HIV infection, ARVs received over the previous 2 years, current medications, viral load suppression status, past medical history, family history of CVD, and tobacco exposure). The participants were categorized into two groups: adolescents (individuals aged 13–17 years) and young adults (individuals aged 18–25 years). BP and resting heart rate were measured with an automated sphygmomanometer (Omron HEM 705 LP; Omron Healthcare Inc., Bannockburn, IL, USA) when the study participants came for refills of their ARVs during September to October 2021. BP was measured in the left arm after resting for at least 5 min in a sitting position, legs uncrossed, with the arm resting on a table and the antecubital fossa at the level of the lower sternum. We selected the appropriate cuff size (small, medium, or large) based on the mid-upper arm circumference. All the nurses involved in measurements of BP underwent a standardized training. Before taking BP measurements, we obtained written informed consent from all participants. Additionally, prior to participation, parents/guardians of the minors (< 18 years) gave written informed consent.

We obtained data on viral load suppression status within 6 months before BP readings. Unsuppressed viral load was defined as viral load > 1,000 copies/mL [13]. Height and weight of participants were measured to the nearest 0.5 cm and 0.1 kg, respectively. We computed body mass index (BMI) as weight (kg) divided by the square of height (m^2^). We adopted categorization of BMI as normal, underweight, overweight, and obese, as per recognized criteria [17]. Tobacco use was defined as any current or previous use. Family history of CVD was defined as a first-degree relative with a history of heart attack, stroke, or HBP.

The assessment of HBP was based on the updated American Academy of Pediatrics definitions of BP categories and stages for individuals aged ≥ 13 years [18]. Accordingly, BP categories were defined as normal BP (< 120/ < 80 mmHg), elevated BP (120/ < 80 to 129/ < 80 mmHg), stage 1 HTN (130/80 to 139/89 mmHg), and stage 2 HTN (≥ 140/90 mmHg). Accordingly, participants with elevated BP or HTN (systolic BP ≥ 120 mmHg or diastolic BP ≥ 80 mmHg) were considered to have HBP. Participants on antihypertensive medication at the time of the survey were also considered to have HBP, regardless of measured BP.

### Data management and statistical analysis

Data were entered by the data collectors using Open Data Kit enabled database. Data were exported to Microsoft Excel (Microsoft Corp., Redmond, WA, USA) and all statistical analyses were performed using Stata ver. 15.0 (Stata Corp., College Station, TX, USA).

We categorized all variables and summarized them using frequencies. Additionally, normally distributed continuous variables, including age, resting heart rate, and BMI were summarized using means ± standard deviation, while non-normally distributed variables (e.g., duration of HIV infection) were summarized using medians and interquartile range (IQR). Associations between HBP and potential associated factors were assessed using modified Poisson regression, reporting prevalence ratios as our measures of association. We chose modified Poisson regression rather than logistic regression due to the high prevalence of our outcome, HBP [19]. All variables demonstrating an association at a *P* < 0.2 significance level were included in the multivariable models. We also adjusted the final model for known confounders, including duration of HIV, HIV viral load, sex, and self-reported family history of CVD. Variables in the final model with a *P* < 0.05 were considered statistically significant.

## Results

### Demographic and clinical characteristics of study participants

We reviewed medical records of 1,045 HIV-infected persons aged 13 to 25 years from nine health facilities in the Rwenzori region, western Uganda. Of the 1,045 participants enrolled, 68% were female (Table 1). The mean age of the participants was 20 ± 3.8 years and 29% were adolescents. The median duration of HIV infection was 6 years (IQR, 3–13 years). Most participants (83%) had suppressed or undetectable viral loads. Nearly all participants (97%) had never smoked. Two-thirds (64%) had normal BMI, and 84% had never consumed alcohol.

**Table 1:** Sociodemographic and clinical characteristics of study participants (*n* = 1,045)

Characteristic	No. (%)
Female sex	714 (68)
Age (yr)^a)^	
13–17	305 (29)
18–25	739 (71)
Level of education	
None	82 (8)
Primary	596 (57)
Secondary	275 (26)
Tertiary	92 (9)
Mode of acquisition	
Vertical transmission	542 (52)
Sexual transmission	503 (48)
Viral load	
Undetectable	186 (18)
Suppressed	682 (65)
Unsuppressed^b^^)^	177 (17)
Family history of cardiovascular disease	
No	889 (85)
Yes	156 (15)
Tobacco use	
Never smoked	1,014 (97)
Current or past smoker	31 (3)
Alcohol use	
Never	882 (84)
Current or past drinker	163 (16)
Body mass index^c^^)^	
Underweight	195 (19)
Normal	664 (64)
Overweight	159 (15)
Obese	27 (3)
Duration since diagnosis of HIV (yr)^d)^	
< 5	432 (41)
5–10	257 (25)
> 10	356 (34)
Duration on antiretroviral therapy (yr)	
< 5	449 (43)
5–10	329 (31)
> 10	267 (26)
Resting heart rate (beats/min)^e)^	
≤ 76	588 (56)
> 76	457 (44)

^a)^Mean ± standard deviation, 20 ± 3.8 years (adolescent group, 13–17 years; young adult group, 18–25 years)

^b)^Defined as > 1,000 copies/mL

^c)^Mean ± standard deviation, 21.8 ± 2.8 kg/m^2^ (underweight, < 18.5 kg/m^2^; normal, 18.5 to 25 kg/m^2^; overweight, 25 to 30 kg/m^2^; obese, ≥ 30 kg/m^2^)

^d)^Median duration of HIV, 6 years (interquartile range, 3–13 years). ^e)^Mean ± standard deviation, 76 ± 11 beats/min

### Prevalence of high blood pressure among HIV-infected young persons

**Fig. 2:**
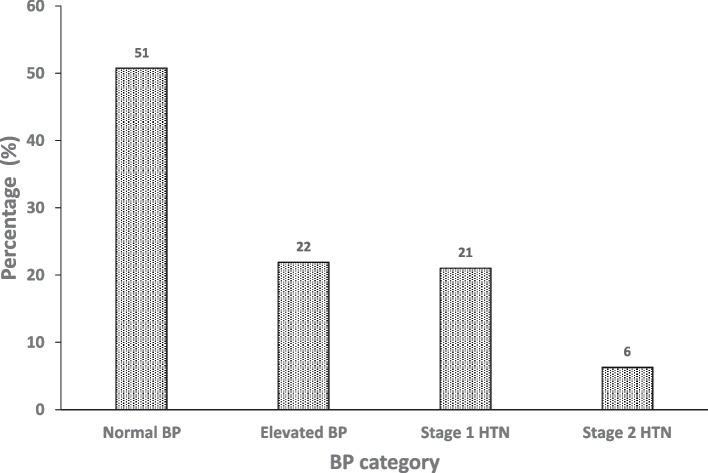
Proportions of blood pressure (BP) categories among HIV-infected young persons aged 13 to 25 years in Rwenzori region, Uganda, from September to October, 2021 (*n* = 1,045). HTN, hypertension

Half of the participants (*n* = 530, 51%) had normal BP and 515 (49%) had HBP. In total, 229 (22%) had elevated BP, 220 (21%) had stage 1 HTN, and 66 (6%) had stage 2 HTN (Fig. 2). Only three participants were already on medications for HBP.

### Sociodemographic and clinical factors associated with high blood pressure among HIV-infected young persons

In multivariable analysis, older age, history of tobacco smoking, and higher resting pulse rate were significantly associated with HBP (Table 2). The prevalence of HBP was 21% higher in the age group of 18 to 25 years compared to 13 to 17 years (adjusted prevalence ratio [aPR], 1.21; 95% confidence interval [CI],1.01–1.44), 41% higher among those with a current or past history of smoking compared to nonsmokers, and 15% higher among those with high resting heart rates (aPR, 1.15; 95% CI, 1.01–1.32; heart rate, > 76 beats/min vs. ≤ 76 beats/min).

**Table 2:** Sociodemographic and clinical factors associated with high blood pressure among HIV-infected young persons aged 13 to 25 years in Kabarole and Kasese districts, western Uganda, from September to October 2021

Characteristic	Blood pressure		Univariable analysis		Multivariable analysis	
	High (*n* = 530)	Normal (*n* = 515)	cPR (95% CI)	*P*-value	aPR (95% CI)	*P*-value
Sex						
Female	352 (68)	362 (68)	Ref		Ref	
Male	163 (32)	168 (32)	1.00 (0.83–1.22)	0.991	1.09 (0.95–1.27)	0.221
Age (yr)						
13–17	125 (24)	181 (34)	Ref		Ref	
18–25	390 (76)	349 (66)	1.29 (1.06–1.58)	0.013	1.21 (1.01–1.44)	0.035
Level of education					-	-
None	38 (7)	44 (8)	Ref			
Primary	279 (54)	317 (60)	1.01 (0.72–1.42)	0.953		
Secondary	145 (28)	130 (25)	1.14 (0.80–1.63)	0.479		
Tertiary	53 (10)	39 (7)	1.24 (0.82–1.89)	0.306		
Mode of acquisition						
Vertical transmission	249 (48)	299 (56)	Ref		Ref	
Sexual transmission	266 (52)	231 (44)	1.18 (0.99–1.40)	0.063	1.17 (0.97–1.43)	0.103
Viral load						
Undetectable	95 (19)	91 (17)	Ref		Ref	
Suppressed	442 (86)	426 (80)	1.00 (0.79–1.24)	0.974	1.01 (0.86–1.19)	0.877
Unsuppressed	73 (14)	104 (20)	0.81 (0.60–1.10)	0.170	0.84 (0.67–1.04)	0.115
Family history of cardiovascular disease						
No	434 (84)	455 (86)	Ref		Ref	
Yes	81 (16)	75 (14)	1.06 (0.84–1.35)	0.611	1.02 (0.86–1.19)	0.820
Tobacco use						
Never smoked	493 (96)	521 (98)	Ref		Ref	
Current or past smoker	22 (4)	9 (2)	1.46 (0.95–2.24)	0.083	1.41 (1.08–1.83)	0.011
Alcohol use						
Never	424 (82)	458 (86)	Ref		Ref	
Current or past drinker	91 (18)	72 (14)	1.16 (0.93–1.46)	0.195	1.05 (0.89–1.25)	0.553
Body mass index (kg/m^2^)						
< 18.5	80 (16)	115 (22)	Ref		Ref	
18.5–25	343 (67)	321 (61)	0.79 (0.62–1.02)	0.063	0.86 (0.71–1.24)	0.109
25–30	75 (15)	84 (16)	0.84 (0.59–1.18)	0.310	0.89 (0.74–1.07)	0.221
≥ 30	17 (3)	10 (2)	1.59 (0.72–3.53)	0.253	1.16 (0.86–1.57)	0.322
Duration since diagnosis of HIV (yr)						
< 5	218 (42)	214 (40)	Ref		Ref	
5–10	127 (25)	130 (25)	0.96 (0.70–1.31)	0.790	1.09 (0.92–1.28)	0.320
> 10	170 (33)	186 (35)	0.90 (0.68–1.19)	0.449	1.19 (0.97–1.17)	0.094
Duration on antiretroviral therapy (yr)					-	-
< 5	224 (44)	225 (43)	Ref			
5–10	153 (30)	176 (33)	0.93 (0.66–1.15)	0.503		
> 10	138 (27)	129 (24)	1.04 (0.084–1.28)	0.744		
Resting heart rate (beats/min)^e)^						
≤ 76	273 (53)	315 (59)	Ref		Ref	
> 76	242 (47)	215 (41)	1.30 (1.02–1.66)	0.036	1.15 (1.01–1.32)	0.044

*cPR* Crude prevalence ratio, *CI* Confidence interval, *aPR* Adjusted prevalence ratio, *Ref* Reference category

## Discussion

HBP was detected in nearly half of the HIV-infected persons aged 13 to 25 years in the Rwenzori region, western Uganda. Among the participants with HBP, close to half (45%) had elevated BP. HBP was significantly associated with increasing age, history of tobacco use, history of alcohol use, and high resting heart rate. Overall, these findings highlight a high burden of HBP among young PLHIV in this region. The results indicate the need for integrating screening and management of HTN and other CVDs into care not only for older but also for younger persons with HIV infection in the region.

There is a paucity of data from sub-Saharan African countries on the prevalence of HBP in adolescents and young PLHIV. The prevalence of HBP in this survey was higher than the prevalence estimates reported (ranging from 10%–40%) in other studies done on general populations of adolescents and young adults in Africa [20–23]. Of note, the prevalence estimates reported in young populations in developing countries have been considerably higher than those reported in high-income countries [24]. This supports the previous notion that the prevalence of HBP is increasing faster in low-income countries than high-income countries, possibly because of increasing urbanization, changes in dietary lifestyle, and/or social stressors [25].

In our study, the prevalence of HBP among HIV-infected young persons aged 13 to 25 years (49%) was higher than that reported in the general population of non-HIV-infected young persons (12–24 years, 40%) in East Africa (Uganda and Tanzania) [20] as well as in the United States [6]. The higher prevalence of HBP among PLHIV may be attributable to a complex interplay of both common risk factors for HTN, such as age, visceral adiposity and/or BMI, smoking, and HIV-related factors [26–28]. However, the diagnostic thresholds for HBP have also been recently lowered [29]. As as result of this change, it is expected that more recent studies will identify a somewhat higher prevalence of HBP than earlier studies implemented before the change. However, it is worth noting that the adoption of the US 2017 new classification of HBP, including HTN, has varied across nations. For instance, the 2018 Korean Society of HTN guidelines, and the 2018 Chinese HTN guidelines still define HTN as systolic blood pressure ≥ 140 mmHg or diastolic blood pressure ≥ 90 mmHg [30, 31]. Regardless of the reasons, our findings support the prioritization of PLHIV for screening and management of HBP to reduce the risk for CVD and poor outcomes.

History of tobacco use, which is a recognized risk factor for CVD [32], was also associated with HBP in this study. Although the use of tobacco was rare (< 10%), these findings highlight a need to reinforce prevention strategies for tobacco abuse among HIV-infected adolescents and young adults to reduce the risk for CVD as well as other smoking-related health hazards (e.g., lung cancer).

In the current survey, increasing resting heart rate was associated with HBP. This is consistent with findings from other studies done among adolescents and young adults in general populations [33–35]. Elevated resting heart rate, like HBP, is an independent risk factor for CVD [35, 36]. It has also been hypothesized that the effect of elevated resting heart rate on adverse CVD outcomes, including mortality, might be mediated through HBP [37]. Thus, persons with both HBP and elevated heart rates could be at increased risk of adverse CVD outcomes. This finding has implications for risk stratification for PLHIV who may be at increased risk for adverse CVD outcomes. HIV care providers should consider such individuals with elevated heart rates for timely interventions, such as optimizing BP control, and routine screening for CVD for early detection and management.

Findings in the current study highlight a need to incorporate programs for screening and management of HBP among all persons living with HIV, including young persons, to enable prompt detection and timely management of HBP including HTN. In resource-limited settings such as Uganda, this NCD/HIV care integration may be possible through leveraging the already existing HIV clinic infrastructure, including human resources for health available for HIV/NCD screening, management, and follow-up of patients [38]. However, successful integrated care processes may require addressing other known structural barriers, including unavailability of reliable blood pressure machines, unreliable NCD drug supply chain, outdated nonefficacious drugs, training and retraining of the healthcare workforce, and reducing long wait times at HIV clinics [39, 40]. Given the high prevalence of HBP in this young population, programs for integration of HIV and NCD care should also consider including young persons, to prevent future CVD epidemics and related adverse cardiovascular events.

Our findings are subject to limitations. First, the cross-sectional design limits us from drawing causal inferences from the observed associations. Second, the utilization of a single clinic visit BP reading may have led to some “white coat hypertension” [41], and potentially overestimated the prevalence of HBP in this survey. Follow-up longitudinal studies employing repeated BP measurements, will be required to assess the burden of sustained HBP and its clinical implications, including the incidence of CVD, in this young population of persons infected with HIV. Third, we did not assess secondary causes of HBP, including renal diseases and endocrine disorders. This may potentially have led to residual confounding from factors that we did not include in our analyses. Finally, this study was conducted in one region of western Uganda and findings from this study may not be generalizable to other regions outside the Rwenzori region. Further studies should be undertaken to better understand the burden of HBP in other regions of Uganda.

## Conclusions

The prevalence of HBP among young HIV-infected Ugandans in this survey was high. Nearly half of the HIV-infected adolescents and young adults surveyed had HBP. Traditional risk factors (increasing age, elevated resting heart rate, and history of tobacco use) were associated with HBP. These data highlight a high burden of HBP in young HIV-infected populations. In order to prevent future CVD epidemics, there is a need to design interventions targeted towards the prevention and control of HBP in HIV-infected persons. Such interventions should also target young persons who were initially considered to be at low risk for CVDs.

## Data Availability

The datasets, upon which our findings are based, belong to the Uganda Public Health Fellowship Program. For confidentiality reasons the datasets are not publicly available. However, the data sets can be availed upon reasonable request from the corresponding author and with permission from the Uganda Public Health Fellowship Program.

## Abbreviations

aPR: Adjusted prevalence ratio
ART: Antiretroviral therapy
ARV: Antiretroviral drug
Baylor Uganda: Baylor College of Medicine Children’s Foundation-Uganda
BMI: Body mass index
BP: Blood pressure
CI: Confidence interval
cPR: Crude prevalence ratio
CVD: Cardiovascular disease
HBP: High blood pressure; HC: Health Center; HOSP: Hospital; HTN: Hypertension
IQR: Interquartile range
NCD: Noncommunicable disease
PEPFAR: President’s Emergency Plan for AIDS Relief; PLHIV: People living with HIV
Ref: Reference category
RRH: Regional Referral Hospital
